# The prevalence and genotype distribution of human papillomavirus among women in Guangxi, southern China

**DOI:** 10.1186/s13027-022-00431-5

**Published:** 2022-04-21

**Authors:** Liuting Wei, Liping Ma, Lingyan Qin, Zhihu Huang

**Affiliations:** Department of Clinical Laboratory, Minzu Hospital of Guangxi Zhuang Autonomous Region, Affiliated Minzu Hospital of Guangxi Medical University, Nanning, 530001 Guangxi Zhuang Autonomous Region China

**Keywords:** Human papillomavirus, Infection rate, Genotype distribution, Guangxi

## Abstract

**Background:**

Human papillomavirus is a primary cause of cervical cancer and genital warts. HPV vaccine can prevent high-grade cervical lesions as well as cervical cancer. The aim of this study was to analyze the prevalence and genotype distribution of human papillomavirus among women in Guangxi before and after the HPV vaccine was approved for use in China.

**Methods:**

From January 2016 to May 2021, 41,140 women were tested for HPV infection. HPV genotyping included 15 high-risk HPV (HR-HPV) and 6 low-risk HPV (LR-HPV) genotypes. Total prevalence, annual trend, and specific age group prevalence and genotype distribution were analyzed.

**Results:**

The overall HPV infection rate was 18.10% among Guangxi women self-referred to clinic for gynecologic problems in southern China. During 2016–2018, the prevalence of HPV infection showed an upward trend, from 18.21% in 2016 to 21.99% in 2018, and later it showed a downward trend, from 18.35% in 2019 to 12.26% in May 2021. Pure HR-HPV genotypes (14.36%) were found in more infections than pure LR-HPV genotypes (2.77%) and mixed genotypes (0.97%). Two peaks of HPV infection were found in the ≤ 25 years (22.94%) and 56–65 years (21.25%) groups. The six most prevalent HR-HPV genotypes were HPV 52 (4.06%), 16 (2.70%), 58 (2.24%), 51 (1.87%), 39 (1.52%), and 53 (1.52%). The three most prevalent LR-HPV genotypes were HPV 6 (1.31%), CP8304 (1.01%), and 11 (0.82%). Infection with a single HR-HPV genotype was the most common type of infection, with an overall infection rate of 12.30%. Infection with two HPV genotypes was the most common multiple HR-HPV infection type, with an infection rate of 2.35%.

**Conclusions:**

The cervical HPV infection rate of women in Guangxi is very high, and there is significant age specificity. There is a need to increase HPV vaccination of young people and the screening of middle-aged and elderly people.

## Introduction

Human papillomavirus (HPV) infection is the main causative factor of cervical cancer; specifically, persistent infection with high-risk HPV (HR-HPV) genotypes. Studies have confirmed that more than 80% of women have had at least one HPV infection [1] and that cervical cancer morbidity and mortality are increasing—it is now the second most common female cancer and cause of death [2].

American Society of Colposcopy and Cervical Pathology (ASCCP) [3] issued a new consensus guideline for abnormal cervical cancer screening tests and risk-based management of cancer precursors in April 2020. It further clarifies the importance of HR-HPV 16/18 detection and infection in risk assessment, and reflects that HR-HPV 16/18 plays an important role in cervical cancer screening, management of abnormal cytology, and follow-up monitoring after cervical cancer treatment. The guidelines point out that if HPV is positive, cytology is required regardless of the HPV genotype. However, HPV16/18 positive has a higher risk of cervical intraepithelial neoplasia grade 3 (CIN3) and occult cancer, so even if the cytology test result is negative, it is necessary to have a colposcopy.

The bivalent HPV vaccine (which protects against HPV 16/18) was approved in China in July 2016, and the quadrivalent vaccine (HPV 6/11/16/18) and nine‑valent vaccine (HPV 6/11/16/18/31/33/45/52/58) were introduced in May 2017 and April 2018, respectively. This study analyzed the status of HPV infection in Guangxi over the past 6 years and examined the trend of HPV infection and genotype distribution. The purpose of this work was to generate information that would allow improvements in the performance of the corresponding healthcare work.

## Materials and methods

### Study population

The study population consisted of women who attended the gynecological clinic of Minzu Hospital in the Guangxi Zhuang Autonomous Region from January 2016 to May 2021. Reasons for their HPV testing include gynecological exams, urethritis, vaginitis, irregular vaginal bleeding, cervicitis, and condyloma acuminatum undiagnosed abdominal pain. The age of the patients ranged from 14 to 87 years old. Information about their HPV test results was collected retrospectively.

### Cervical sample collection

A speculum was used to expose the cervix, and a dry cotton swab was used to remove cervical secretions. A cervical brush was placed in the cervical canal and unidirectionally rotated four to five times to ensure that sufficient epithelial cells were obtained. The cervical brush was then placed into a sample preservation tube with preservation solution and the cap tightened. The sample tubes were compatible with the HPV genotyping test kit of Chaozhou Hybribio Biochemical Co., Ltd. (China). In accordance with the manufacturer’s instructions, all samples were sent to the laboratory within 24 h and stored at 4 °C, and HPV testing was performed within one week.

### HPV genotyping

All collected samples were genotyped for HPV using the HPV Genotyping Test Kit (polymerase chain reaction (PCR) + flow-through hybridization; Chaozhou Hybribio Biochemical Co., Ltd., Guangdong, China). The test has been approved by the State Food and Drug Administration (Permit Number: 0041040) and can detect 21 HPV genotypes: 6, 11, 16, 18, 31, 33, 35, 39, 42, 43, 44, 45, 51, 52, 56, 58, 59, 66, 68, and CP8304.

DNA extraction, amplification, and hybridization and interpretation of the results were carried out in accordance with the instructions of the kit. DNA was extracted using an automated nucleic acid extraction apparatus (HBNP 4801A, Chaozhou Hybribio Biochemical Co., Ltd., Guangdong,China). An ABI 9700 PCR instrument was used to perform gene amplification. An automatic nucleic acid molecule rapid hybridization instrument (HBHM-3000S) was used for reverse dot hybridization. Negative and positive controls were established throughout the experiment. At the same time, internal quality control and external quality evaluation were carried out, and the results were in line with the requirements of the laboratory.

### Statistical analysis

The data were added to an Excel spreadsheet, filtered, and stratified by age (≤ 25 years, 26–35 years, 36–45 years, 46–55 years, 56–65 years, and ≥ 66 years). Analyses were conducted with SPSS version 20.0. HPV infection rate differences across years and age groups were calculated using the chi-square test. *P* < 0.05 was considered statistically significant. GraphPad Prism 5.0 software was used for drawing.

## Results

### The overall HPV infection rate

This study included 41,140 women who attended Minzu Hospital in the Guangxi Zhuang Autonomous Region. The overall HPV infection rate was 18.10% (7588/41,926) during the study period of January 2016 to May 2021 (Table 1). The infection rate with pure LR-HPV genotypes was 2.77% (1162/41,926), with pure HR-HPV genotypes was 14.36% (6019/41,926), and with a combination of both was 0.97% (407/41,926). With the LR-HPV and HR- HPV infection rates found to be 3.74% (1569/41,926) and 15.33% (6426/41,926), respectively, HR-HPV infection has an obvious advantage. The HPV infection rates were significantly different among the different years (χ^2^ = 238.352, *df* = 5, *P* < 0.001). The highest overall HPV infection rate was found to be in 2018 (21.99%), while the lowest HPV infection rate was found to be in 2021 (12.26%). During 2016–2018, the prevalence of HPV infection showed an upward tendency, and it showed a downward tendency from 2019 to May 2021.

**Table 1:** Trends of HPV infection rates from 2016 to 2021

HPV subtype	2016		2017		2018		2019		2020		2021		Total	
	n	%	n	%	n	%	n	%	n	%	n	%	n	%
Pure LR HPV	123	2.19	190	2.77	299	3.67	269	2.83	232	2.74	49	1.48	1162	2.77
Pure HR HPV	812	14.45	1036	15.10	1427	17.51	1422	14.97	987	11.64	335	10.09	6019	14.36
Mixed infection	88	1.57	143	2.08	66	0.81	52	0.55	35	0.42	23	0.69	407	0.97
Total infection	1023	18.21	1369	19.95	1792	21.99	1743	18.35	1254	14.80	407	12.26	7588	18.10
Total tests	5620		6864		8148		9498		8477		3319		41,926	

### Infection rates of different age groups

All participants were divided into six groups according to their age (≤ 25 years, 26–35 years, 36–45 years, 46–55 years, 56–65 years, and ≥ 66 years). The HPV infection rates were significantly different among the age groups (χ^2^ = 145.481, *df* = 5, *P* < 0.001). Two peaks of HPV infection were found in the ≤ 25 years (22.94%) and 56–65 years (21.25%) groups (Table 2). Women aged 0–45 years had the highest infection rates in 2018, and then showed a downward trend (Table 3). The 26–35 years group made up the largest proportion of screened and infected participants, accounting for 34.87% of the overall positive population.

**Table 2:** HPV infection rates in different age groups

Age group (years)	Total (n)	Positive (n)	%^a^	%^b^
≤ 25	6208	1424	22.94	18.77
26–35	15,042	2646	17.59	34.87
36–45	11,124	1798	16.16	23.70
46–55	7176	1230	17.14	16.20
56–65	1807	384	21.25	5.06
≥ 66	569	106	18.63	1.40
Total	41,926	7588	18.10	100.00

%^a^: Infection rates in different age groups

%^b^: Proportion of positives in different age groups

**Table 3:** HPV infection rates in different age groups in each year

Age group	2016			2017			2018			2019			2020			2021		
(years)	Total	Positive	%	Total	Positive	%	Total	Positive	%	Total	Positive	%	Total	Positive	%	Total	Positive	%
≤ 25	809	206	25.46	858	215	25.06	980	272	27.76	1630	351	21.53	1425	282	19.79	506	98	19.37
26–35	2147	362	16.86	2570	498	19.38	3075	653	21.24	3370	590	17.51	2780	423	15.22	1100	120	10.91
36–45	1651	252	15.26	1987	338	17.01	2302	499	21.68	2295	386	16.82	2043	251	12.29	846	72	8.51
46–55	798	140	17.54	1148	212	18.47	1403	283	20.17	1611	307	19.06	1593	204	12.81	623	84	13.48
56–65	169	57	33.73	223	73	32.74	302	68	22.52	435	83	19.08	500	83	16.60	178	20	11.24
≥ 66	46	6	13.04	78	33	42.31	86	17	19.77	157	26	16.56	136	11	8.09	66	13	19.70

### HPV genotype distribution

**Fig. 1:**
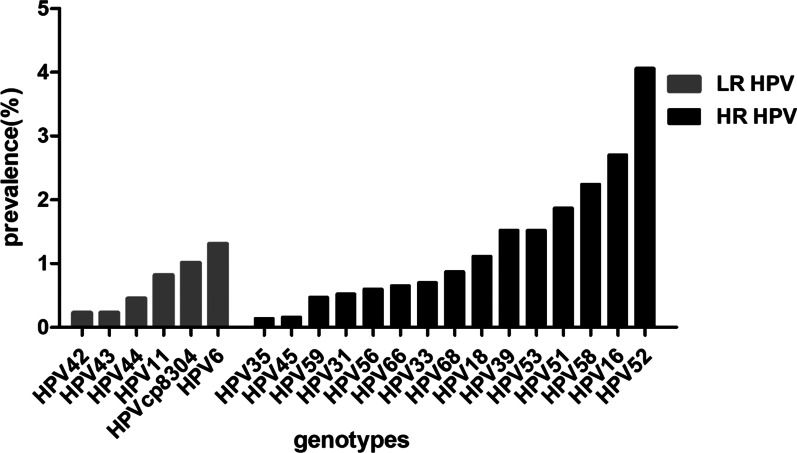
Overall prevalence and distribution of HPV (HR-HPV and LR-HPV)

**Fig. 2:**
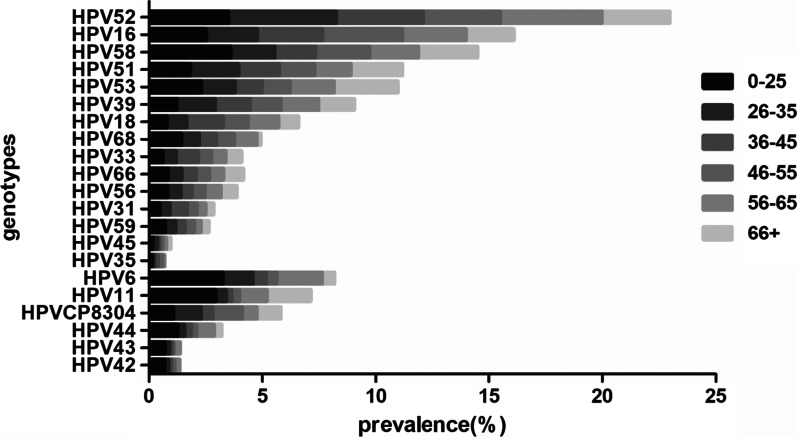
Distribution of subtypes in different age groups

From January 2016 to May 2021, the six most common HR-HPV genotypes were HPV 52, 16, 58, 51, 39, and 53 (Fig. 1) at overall frequencies of 4.06%, 2.70%, 2.24%, 1.87%, 1.52%, and 1.52%, respectively (Table 4), followed by HPV 18/68/33/66/56/31/59/45/35. The trend was the same every year. In addition, the most common LR-HPV genotypes were HPV 6 (1.31%), CP8304 (1.01%), and 11 (0.82%). HPV 6, 11, and 58 infections were mainly concentrated in the ≤ 25 years group (3.24%, 2.92%, and 3.95%, respectively; Fig. 2). HPV 52 was the most common genotype in the 26–35 (4.62%), 36–45 (5.36%), 56–66 (4.48%), and ≥ 66 (2.99%) years groups (Table 5). Whereas HPV 16 was the most common genotype in the 46–55 years group (3.53%). The highest HPV 18 infection rate was found in the 36–45 years group (1.60%). HPV 35/45/31/33 showed lower infection rates in all age groups.

**Table 4:** Summary of HPV genotype infections from January 2016 to May 2021

HPV genotype	2016	2017	2018	2019	2020	2021	Total
	n (%)	n (%)	n (%)	n (%)	n (%)	n (%)	n (%)
HPV 6	57 (1.01)	75 (1.09)	120 (1.47)	139 (1.46)	113 (1.33)	37 (1.11)	541 (1.31)
HPV 11	35 (0.62)	57 (0.83)	69 (0.84)	81 (0.85)	79 (0.93)	16 (0.48)	337 (0.82)
HPV 42	5 (0.09)	27 (0.39)	34 (0.42)	15 (0.12)	15 (0.18)	2 (0.06)	98 (0.23)
HPV 43	9 (0.16)	29 (0.42)	22 (0.27)	15 (0.12)	17 (0.20)	3 (0.09)	95 (0.23)
HPV 44	12 (0.21)	58 (0.84)	56 (0.69)	30 (0.32)	24 (0.28)	8 (0.24)	188 (0.45)
HPVcp8304	89 (1.58)	110 (1.60)	98 (1.20)	68 (0.72)	39 (0.46)	14 (0.42)	418 (1.01)
HPV 16	152 (2.70)	198 (2.88)	242 (2.97)	256 (2.70)	212 (2.50)	72 (2.17)	1132 (2.70)
HPV 18	68 (1.21)	91 (1.33)	110 (1.35)	108 (1.14)	68 (0.80)	22 (0.66)	467 (1.11)
HPV 31	31 (0.55)	45 (0.66)	61 (0.75)	48 (0.51)	28 (0.33)	7 (0.21)	220 (0.52)
HPV 33	43 (0.77)	53 (0.77)	70 (0.86)	77 (0.81)	38 (0.45)	12 (0.36)	293 (0.70)
HPV 35	13 (0.23)	12 (0.17)	14 (0.17)	11 (0.12)	3 (0.04)	5 (0.15)	58 (0.14)
HPV 39	94 (1.67)	118 (1.72)	130 (1.60)	154 (1.62)	95 (1.12)	46 (1.39)	637 (1.52)
HPV 45	7 (0.12)	15 (0.22)	12 (0.15)	17 (0.18)	11 (0.13)	3 (0.09)	65 (0.16)
HPV 51	93 (1.65)	188 (2.74)	184 (2.26)	169 (1.78)	122 (1.44)	29 (0.87)	785 (1.87)
HPV 52	224 (3.99)	317 (4.62)	437 (5.36)	412 (4.34)	226 (2.67)	86 (2.59)	1702 (4.06)
HPV 53	90 (1.60)	107 (1.56)	139 (1.7 1)	181 (1.91)	91 (1.09)	29 (0.87)	637 (1.52)
HPV 56	36 (0.64)	48 (0.70)	72 (0.88)	55 (0.58)	29 (0.34)	9 (0.27)	249 (0.60)
HPV 58	121 (2.15)	178 (2.59)	209 (2.57)	205 (2.16)	168 (1.98)	58 (1.75)	939 (2.24)
HPV 59	21 (0.37)	36 (0.52)	47 (0.58)	50 (0.53)	32 (0.38)	10 (0.30)	196 (0.47)
HPV 66	32 (0.57)	60 (0.87)	60 (0.74)	52 (0.55)	55 (0.65)	12 (0.36)	271 (0.65)
HPV 68	41 (0.73)	79 (1.15)	105 (1.29)	72 (0.76)	46 (0.54)	23 (0.69)	366 (0.87)
Total	1273	1901	2291	2215	1511	503	9694

%: the ratio of the number of HPV genotype infections to the total number of participants, N = 41,926

**Table 5:** Distribution of HPV genotypes in different age groups

HPV genotypes	≤ 25	26–35	36–45	46–55	56–65	≥ 66
	n (%)	n (%)	n (%)	n (%)	n (%)	n (%)
HPV6	201 (3.24)	201 (1.34)	66 (0.59)	34 (0.47)	36 (1.99)	3 (0.53)
HPV11	181 (2.92)	71 (0.47)	27 (0.24)	25 (0.35)	22 (1.22)	11 (1.93)
HPV42	42 (0.68)	26 (0.17)	15 (0.13)	11 (0.15)	4 (0.22)	0 (0.00)
HPV43	44 (0.71)	27 (0.18)	14 (0.13)	5 (0.07)	5 (0.28)	0 (0.00)
HPV44	78 (1.26)	46 (0.31)	31 (0.28)	17 (0.24)	14 (0.77)	2 (0.35)
HPVCP8304	67 (1.08)	182 (1.21)	59 (0.53)	92 (1.28)	12 (0.66)	6 (1.05)
HPV16	156 (2.51)	342 (2.27)	318 (2.86)	253 (3.53)	51 (2.82)	12 (2.11)
HPV18	49 (0.79)	131 (0.87)	178 (1.60)	80 (1.11)	24 (1.33)	5 (0.88)
HPV31	29 (0.47)	67 (0.45)	85 (0.76)	30 (0.42)	7 (0.39)	2 (0.35)
HPV33	37 (0.60)	91 (0.60)	107 (0.96)	43 (0.60)	11 (0.61)	4 (0.70)
HPV35	7 (0.11)	16 (0.11)	20 (0.18)	13 (0.18)	2 (0.11)	0 (0.00)
HPV39	75 (1.21)	255 (1.70)	171 (1.54)	97 (1.35)	30 (1.66)	9 (1.58)
HPV45	10 (0.16)	31 (0.21)	11 (0.10)	9 (0.13)	3 (0.17)	1 (0.18)
HPV51	113 (1.82)	320 (2.13)	198 (1.78)	112 (1.56)	29 (1.60)	13 (2.28)
HPV52	217 (3.50)	715 (4.75)	427 (3.84)	245 (3.41)	81 (4.48)	17 (2.99)
HPV53	143 (2.30)	222 (1.48)	133 (1.20)	88 (1.23)	35 (1.94)	16 (2.81)
HPV56	50 (0.81)	88 (0.59)	54 (0.49)	40 (0.56)	13 (0.72)	4 (0.70)
HPV58	223 (3.59)	293 (1.95)	199 (1.79)	170 (2.37)	39 (2.16)	15 (2.64)
HPV59	44 (0.71)	69 (0.46)	46 (0.41)	30 (0.42)	5 (0.28)	2 (0.35)
HPV66	52 (0.84)	91 (0.60)	70 (0.63)	42 (0.59)	11 (0.61)	5 (0.88)
HPV68	88 (1.42)	119 (0.79)	84 (0.76)	56 (0.78)	18 (1.00)	1 (0.18)
Total	1906	3403	2313	1492	452	128

### Distribution of single and multiple HR-HPV infections

The overall HR-HPV infection rate was 15.33%. For all the HR-HPV positive cases, it was found that infection with a single HR-HPV genotype was the most common type of infection, with an overall infection rate of 12.30% (5,156/41,926; Table 6) that accounted for 80.24% (5,156/6,426) of the total HR-HPV positive cases. Infection with multiple HR-HPV genotypes occurred at a rate of 3.03% (1,270/41,926), accounting for 19.76% of the total HR-HPV positive cases. Among the multiple genotype cases, two genotypes were the most common, and the infection rate was 2.35%. This was 15.33% of the total number of HR-HPV infections and 77.56% (985/1,270) of the total number of multi-genotype infections. Three genotypes in 3.31% of HR-HPV infections, four genotypes in 0.84%, and five or more genotypes in only 0.28%.

**Table 6:** Single-type and MULTIPLE-type HR HPV infection rates

Number of HPV subtype	2016	2017	2018	2019	2020	2021	Total	%^c^	%^d^
Single HPV subtype	714	915	1189	1172	861	305	5156	12.30	80.24
Two HPV subtypes	148	191	237	235	131	43	985	2.35	15.33
Three HPV subtypes	30	56	47	47	25	8	213	0.51	3.31
Four subtypes	6	14	13	16	3	2	54	0.13	0.84
≥ Five HPV subtypes	2	3	7	4	2	0	18	0.04	0.28
Total	900	1179	1493	1474	1022	255	6426	15.33	100.00

%^c^: Infection rate of single or multiple subtypes of HR HPV, N = 41,926

%^d^: Percentage of the total number of high-risk HPV positives, N = 6426

## Discussion

HPV infection plays an important role in the occurrence and development of cervical cancer. Numerous studies have shown that HPV genotypes and infection rates differ among countries and regions [4, 5]. For example, a rate of 24.9% has been reported in Africa, 15.6% in America, 8.3% in Asia, and 6.6% in Europe. In addition, HPV infection rates differ across regions in China [6–8] Here, we analyzed the HPV prevalence trends from 2016 to May 2021 and found that the overall infection rate was 18.10% (Table 1). This is higher than reported in Yunnan (12.9%) [6], Shanghai (17.92%) [8], Xinjiang (14.02%) [9], and Huzhou (15.5%) [10], and lower than reported in Guangzhou (26.0%) [7], Sichuan (23.84%) [11], and Chongqing (19.9%) [12]. In addition to cultural background and living habits, ethnicity may be an important contributing factor to HPV prevalence in different regions. A study of HPV infection among different ethnic groups in Yunnan showed that the prevalence rate among Tibetan women was significantly higher than that among Naxi and Han women [13].

Our findings show that the HPV infection rate varied significantly over time. The HPV infection rate increased year by year before 2018 and was the highest in 2018 (21.99%). The rate then generally declined from 18.35% in 2019 to 12.26% in 2021. Luo et al. [14] showed that the HPV infection rate in southern China was trending down from 2012 to 2018, and that Xinjiang HPV infections peaked in 2014 [9]. It is worth noting that reported infection rates, and thus trends, may be affected by demographic factors (e.g., ethnicity and cultural diversity) and by technical factors, such as sampling strategies, equipment, methods, and the specificity and sensitivity of HPV detection analysis [9, 13–15].

Participants were placed into six groups according to their age (≤ 25 years, 26–35 years, 36–45 years, 46–55 years, 56–65 years, and ≥ 66 years). Two peaks of HPV infection were detected in the ≤ 25 years (22.94%) and 56–65 years (21.25%) groups (Table 2), a finding that is consistent with those of many studies [6, 16, 17]. The sexual activity and immature immune protection of young women, as well as the physiological and immunological disorders related to hormone fluctuations during the menopausal transition of older women, could be reasons for these two peaks. At the same time, although the risk of HPV infection in young people is known to be high, it is temporary and should disappear within 1 or 2 years; hence, HPV infection prevalence gradually decreases with age [9]. Among the women ≤ 45 years of age, the infection rate was highest in 2018, and then decreased year by year (Table 3). In addition, HPV screening was primarily concentrated in the 26–35 years group, which may be related to education level, financial ability, and health awareness; this age group has high health awareness. The older women had less HPV-related knowledge than the younger women, so they were not actively seeking screening as often as the young women. Older women have been shown to have higher cervical cancer detection rates than younger women [18]; therefore, older women should be actively screened for HPV.

Among the participants with HPV infections, high-risk genotypes were predominant. The HR-HPV infection rate was more than four times higher than the LR-HPV infection rate, and infection with pure HR-HPV (14.36%) was higher than that with pure LR-HPV (2.77%) and mixed HPV (0.97%; Table 1). The HR-HPV infection rate varies across continents: 22.1% in Africa, 11.3% in North America, 8.1% in Europe, and 8% in Asia [19]. The HR-HPV infection rate is also different in various regions of China. Our frequency of HR-HPV infection (15.33%) is lower than that previously reported in Fujian (18.68%) [20], Yangzhou (23.56%) [21], and Guangzhou (21.12%) [7], higher than that previously reported in Sichuan (12.6%) [22], Xinjiang (9.72%) [9], and Beijing (7.0%), and similar to that reported in Shanghai (15.56%) [8].

Among the positive cases of HR-HPV infection, single genotype infection was significantly higher than multiple genotype infection. The single genotype infection rate was 12.30%, accounting for 80.24% of HR-HPV infection (Table 5), and the multiple genotype infection rate was 3.03%. Among the multiple infection cases, two genotypes were predominant (2.35%).

The results of this study showed that HPV 52, 16, 58, 51, 39, and 53 were the most common genotypes in the Guangxi Chinese population. This finding is consistent with the results reported in Shanghai [8] and different from those in other Chinese cities, such as Meizhou (HPV 52, 16, 58, 39, 53, and 18) [23], Xinjiang (HPV 16, 52, 58, 53, 31, and 39; HPV 51 ranks tenth among HR-HPV infections) [9], Yunnan (HPV 16, 33, 52, 31, 58, and 39) [13], Beijing (HPV 16, 58,33,52, 18, and 31) [17], and Sichuan (HPV 52, 53, 58, 16, 56, and 39) [11].

Although the main genotypes vary from region to region, HPV 16 and 18 are the most common worldwide, and they account for about 70.9% of cervical cancers [24]. At the same time, patients with recurrence have been shown to have persistent HR-HPV infection, and 74.5% have HPV 16/18 [25]. In our study, the infection rate of HPV 16 was 2.70% and ranked second, and the highest infection rate was found in the 46–55 years group (3.53%). The HPV 18 infection rate was 1.11% and ranked seventh, and the highest infection rate was found in the 36–45 years group (1.60%). Squamous intraepithelial lesions (SILs) are precursors of cervical cancer. A study conducted in Mexican women found that the HPV genotypes common in high-risk SILs are HPV 16, 31, 39, 51, 52, 58, and 59 [26]. Guan et al. showed that the most commonly detected HR-HPV genotypes in women with invasive cancer were HPV 16, 18, and 45 [27]. Furthermore, the main HR-HPV genotypes detected among women with high-grade SILs (HSILs) in Shanghai were HPV 16, 58, 52, and 33 [28]; although, the HPV 52 and 33 infection rates in HSIL cases were significantly lower than those in cases of cervicitis/negative for intraepithelial ledion or malignancy (NILM), atypical squamous cells of undetermined significance (ASC-US), and low-grade SILs (LSIL). However, a study in northeast China showed that women infected with HPV 16, 18, 31, and 33 had a high incidence of HSILs [29]. A limitation of this study is that there was no pathological data available (e.g., cervical cytology and histology results), and thus it is not possible to determine the relationships between HPV genotype and pathology.

Nine-valent vaccines include HPV 6, 11, 16, 18, 31, 33, 45, 52, and 58. In this study, the infection rates of HPV 51, 53, and 39 were significantly higher than those of HPV 33, 31, and 45. This indicates that the nine-valent vaccines do not provide protection against the main infection genotypes (HPV 51, 53, and 39). The related studies can provide data references for vaccine development. HPV 6 and 11 infections were mainly concentrated in the ≤ 25 years group, and infection with these genotypes was found to be low in the other age groups.

## Conclusion

In summary, the HPV infection rate in Guangxi is high (18.10%), it had an upward trend from 2016 to 2018, a downward trend after 2018, and was dominated by single high-risk genotypes. The highest rates of HPV infection were in women ≤ 25 years and 56–65 years. The main genotypes were HPV 52, 16, 58, 51, 39, and 53. The 46–55 years group had the highest HPV 16 infection rate, and the highest HPV 18 infection rate was found in the 36–45 years group.

Given that China is a developing country with a population of 1.4 billion, that the most prevalent HR-HPV genotypes vary across regions, and that there is significant age specificity, understanding HPV genotype distribution is essential for better planning of cervical cancer screening and HPV vaccine development. It is necessary to increase HPV vaccination of young people and the screening of middle-aged and elderly people.

## Data Availability

All data generated or analysed during this study are included in this published article.
